# Engineered Type Six Secretion Systems Deliver Active Exogenous Effectors and Cre Recombinase

**DOI:** 10.1128/mBio.01115-21

**Published:** 2021-07-20

**Authors:** Steven J. Hersch, Linh Lam, Tao G. Dong

**Affiliations:** a Department of Ecosystem and Public Health, University of Calgary, Calgary, Alberta, Canada; b State Key Laboratory of Microbial Metabolism, Joint International Research Laboratory of Metabolic & Developmental Sciences, School of Life Sciences and Biotechnology, Shanghai Jiao Tong University, Shanghai, China; McMaster University

**Keywords:** type six secretion system, protein secretion, genetic editing, Cre recombinase, interspecies interactions, effector, antimicrobial, *Aeromonas dhakensis*, DNA recombination, *Pseudomonas aeruginosa*, T6SS, *Vibrio cholerae*, bactericidal activity, biotechnology, protein engineering

## Abstract

Genetic editing has revolutionized biotechnology, but delivery of endonuclease genes as DNA can lead to aberrant integration or overexpression, leading to off-target effects. Here, we develop a mechanism to deliver Cre recombinase as a protein by engineering the bacterial type six secretion system (T6SS). Using multiple T6SS fusion proteins, Aeromonas dhakensis or attenuated Vibrio cholerae donor strains, and a gain-of-function cassette for detecting Cre recombination, we demonstrate successful delivery of active Cre directly into recipient cells. The most efficient transfer was achieved using a truncated version of PAAR2 from V. cholerae, resulting in a relatively small (118-amino-acid) delivery tag. We further demonstrate the versatility of this system by delivering an exogenous effector, TseC, enabling V. cholerae to kill Pseudomonas aeruginosa. This implies that P. aeruginosa is naturally resistant to all native effectors of V. cholerae and that the TseC chaperone protein is not required for its activity. Moreover, it demonstrates that the engineered system can improve T6SS efficacy against specific pathogens, proposing future application in microbiome manipulation or as a next-generation antimicrobial. Inexpensive and easy to produce, this protein delivery system has many potential applications, ranging from studying T6SS effectors to genetic editing.

## INTRODUCTION

Genetic editing tools have provided incredible resources for DNA manipulation. In addition to the versatile and guided endonuclease, CRISPR-Cas9, other enzymes, such as Cre recombinase, transformed the field of biotechnology (1). Originally from the Escherichia coli P1 bacteriophage, Cre recombines genetic material to remove DNA flanked by *loxP* sites (floxed), and this has been harnessed in diverse organisms, ranging from bacteria to mice (1, 2). However, genetic editing enzymes have limitations, including that they must be introduced into a target cell. This is typically done by transferring DNA encoding the active enzyme, which potentially can cause unwanted mutations by integrating into the target chromosome or overexpressing the enzyme (3–5). Delivering genetic editors as proteins can potentially alleviate these issues by eliminating the possibility of DNA integration while simultaneously allowing for more control over enzyme dosage.

Bacteria encode numerous protein secretion systems, such as the type 3 (T3SS) and type 4 secretion systems (T4SS), which are often virulence factors that pass unfolded effector proteins through a central pore into eukaryotic host cells (6). Several successful endeavors have demonstrated that these secretion systems can be harnessed for delivery of recombinant proteins (7, 8). Another, more recently discovered system is the type six secretion system (T6SS) (9, 10). The T6SS resembles a molecular spear gun and uses physical puncturing to penetrate nearby bacteria or eukaryotic cells in order to deliver protein effectors with various destructive activities (11, 12). Importantly, the toxicity of the system toward prey cells is primarily driven by the effectors, since the damage caused by the puncture itself appears negligible, as demonstrated by effectorless strains constructed in multiple species (13–17).

Rather than passing unfolded proteins through a pore like the T3SS and T4SS, the T6SS mounts effectors onto the spear structure, which permits folding prior to delivery. Effectors can be loaded in the long Hcp protein tube that is thrust forward or mounted on a pointed spearhead comprised of a trimer of VgrG proteins and a sharp PAAR protein tip. Notably, effectors can be noncovalently attached for delivery (cargo effectors) or included as extended domains of the structural proteins, which are termed evolved or specialized effectors. Past attempts at delivering recombinant proteins using T6SSs have revealed key challenges (18) as well as some successes, which include delivery of β-lactamase into eukaryotic cells (19–21) and fusion of two exogenous effectors for secretion (21, 22). Vibrio cholerae encodes one of the best-characterized T6SS, and the T6SS has been particularly well characterized in strain V52, which is a nonpandemic strain with a constitutively active T6SS (9). V. cholerae V52 naturally employs five known effectors to inhibit macrophage or amoebae that phagocytose it or to kill neighboring bacteria, including T6SS^+^ competitors such as Aeromonas dhakensis (23–26). In contrast, some species, such as Pseudomonas aeruginosa, survive attacks by the V. cholerae T6SS, but the mechanism of this resistance remains uncertain (27). The five T6SS effectors of V. cholerae include two evolved effectors, VgrG1 (containing an actin cross-linking domain) and VgrG3 (containing a lysozyme domain) (28, 29). The other three are cargo effectors, VasX, TseL, and TseH, which are loaded onto VgrG2, VgrG1, or PAAR2, respectively (17, 30, 31). Furthermore, the V52 strain is genetically malleable and was previously attenuated by deletion of three non-T6SS toxins, *rtxA*, *hlyA*, and *hapA* (9), rendering it less toxic to eukaryotic cells. This makes it a potentially viable donor for downstream applications of protein delivery into eukaryotic cells or in the context of a host organism.

In this work, we demonstrate that the T6SS can be harnessed by engineering fusion tags that facilitate delivery of active Cre recombinase directly into neighboring recipient cells. We picked Cre recombinase as the delivered exogenous protein for the following reasons. (i) Cre has previously been used to demonstrate engineered T3SS-mediated delivery (32). (ii) The size of Cre (343 amino acids) is comparable to the effector domains of VgrG1 and VgG3. (iii) Cre instigates efficient and specific DNA recombination, allowing for detection in recipient cells. (iv) Cre/*loxP* recombination is commonly used in numerous species; therefore, Cre delivery has immediate potential applications. (v) Cre delivery represents a proof of principle for transfer of active genetic editing enzymes into the cytoplasm of target cells, which establishes the system for future development using targeted endonucleases such as CRISPR-Cas9.

We developed a relatively small (118 amino acids), yet highly efficient, delivery tag that achieves substantial levels of *loxP* site recombination in recipients; this highlights its potential for future genetic editing applications. We further demonstrate the utility of the system by efficiently delivering an exogenous T6SS effector that grants V. cholerae the ability to kill P. aeruginosa, demonstrating the application of the system to studying effector activity or targeting particular bacterial prey.

## RESULTS

### Cre fused to VgrG is secreted in a T6SS-dependent manner.

We hypothesized that evolved VgrG proteins would be most amenable to secreting exogenous proteins due to their natural fusion to effector domains. Specifically, by replacing the effector domain with a protein of interest, toxic effector activity is removed while leaving the natural linker to the VgrG domain intact, which may be important for proper folding and loading onto the T6SS tip. Since V. cholerae V52 has two evolved VgrGs and one of the best-studied T6SSs, we employed it to deliver Cre recombinase cargo in initial T6SS engineering experiments.

**FIG 1:**
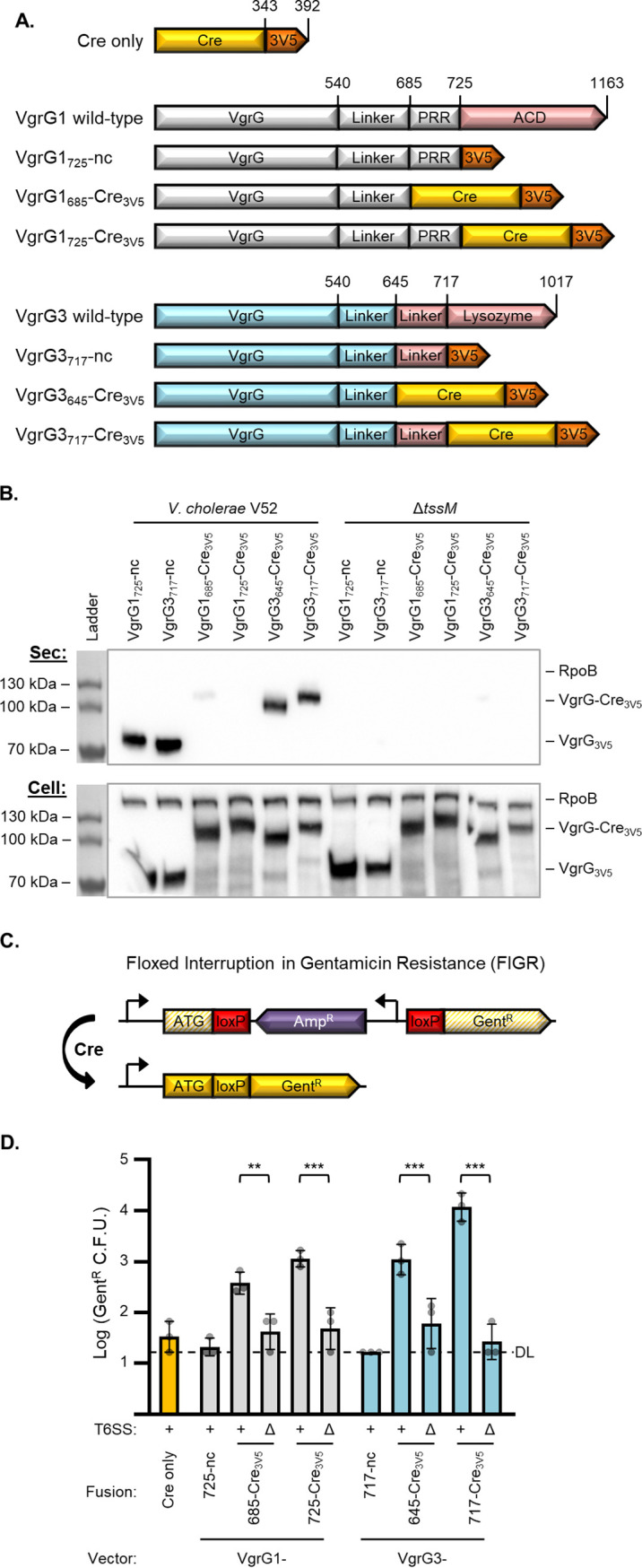
Active VgrG-Cre fusions are secreted and delivered to recipient cells in a T6SS-dependent manner. (A) Depiction (not to scale) showing fusions of Cre recombinase (yellow) and 3× V5 tag (orange) to VgrG1 and VgrG3 of V. cholerae V52. VgrG1 (gray) wild type includes a proline-rich region (PRR) and an actin cross-linking domain (red; ACD). VgrG3 (blue) wild type includes a lysozyme domain (red) with a linker region leading up to the peptidoglycan binding domain beginning at codon 718. nc, no Cre. (B) Western blot showing secretion of VgrG1 and VgrG3 fusions from wild-type V. cholerae V52 but not an equivalent *tssM* mutant. Sec (top) shows secreted fractions, and Cell (bottom) shows cell pellet lysates. In addition to α-V5, α-RpoB antibody was included as a cytoplasm control. Representative of three independent replicates. (C) Depiction of the floxed interruption in gentamicin resistance (FIGR) cassette. An ampicillin resistance cassette (purple), located in the reverse direction and flanked by *loxP* sites (red), interrupts the open reading frame of a gentamicin resistance gene (yellow striped). Exposure to Cre removes the block and allows expression of the gentamicin resistance gene (yellow). Promoters are shown as 90° arrows. Figure is not to scale. ATG, Gent^r^ start codon. (D) Recovery of V. cholerae Gent^r^ CFU (indicative of Cre-mediated FIGR cassette recombination) after delivery from wild-type (+) or Δ*tssM* (Δ) V. cholerae with the indicated Cre fusions to VgrG vectors. DL, detection limit. One-way analysis of variance (ANOVA) with Sidak’s multiple-comparison test comparing wild-type and Δ*tssM* donors with equivalent delivery fusions. Recovery from Δ*tssM* samples were not significantly above Cre only or no Cre (nc) controls. **, *P* < 0.01; ***, *P* < 0.001.

To generate VgrG-Cre fusions, we first inserted *vgrG* genes, truncated prior to their effector domains, into a plasmid vector with a C-terminal 3× V5 tag (Fig. 1A); these effectorless VgrG plasmids served as no-Cre controls. Notably, the remaining linker region of VgrG1 includes a proline-rich region (PRR; codons 686 to 725) where 35% of the residues are proline, including six in a row. VgrG3 also has a linker between the effector domain and the VgrG domain; the conserved domains analysis feature of BLAST (33) revealed that this region could be split into a linker associated with the lysozyme domain (646-717) and another linker region (540-645). Since it was unclear if these linkers were important for T6SS-mediated secretion, we introduced Cre recombinase at two different locations, either maintaining the entire linker (VgrG1_725_-Cre_3V5_ and VgrG3_717_-Cre_3V5_) or removing the PRR or lysozyme-associated linker domains (VgrG1_685_-Cre_3V5_ and VgrG3_645_-Cre_3V5_, respectively) (Fig. 1A).

We electroporated the VgrG-Cre plasmids into the V. cholerae V52 strain and tested secretion by Western blotting for the V5 antigen. Both no-Cre controls were secreted from wild-type bacteria but not from an equivalent T6SS-inactive (Δ*tssM*) mutant (Fig. 1B). These data suggest T6SS-dependent release of these truncated VgrGs, since the proteins were fully expressed in the Δ*tssM* mutant (whole-cell fractions), and no cell lysis was observed (measured as RNA polymerase beta subunit, RpoB, in the secreted fractions). Moreover, both VgrG3-Cre fusions exhibited prominent T6SS-dependent secretion (Fig. 1B).

In contrast, the VgrG1-Cre fusions were only slightly detectable in the secreted fractions (Fig. 1B). This suggests that VgrG1-Cre is either unstable in the extracellular milieu or is secreted much less than VgrG3-Cre. To test if VgrG1-Cre was secreted at all, we tested its ability to deliver the cargo effector, TseL. Normally TseL binds to VgrG1 for delivery and a V52 *vgrG1* mutant does not kill a sensitive strain lacking the TseL immunity gene, *tsiV1* (30, 31). The VgrG1_725_-Cre_3V5_ fusion was able to complement TseL delivery in a Δ*vgrG1* strain (see Fig. S1 in the supplemental material), suggesting that VgrG1_725_-Cre_3V5_ can bind to TseL and be secreted into neighboring cells. We therefore continued to examine the VgrG1-Cre fusions in further assays.

10.1128/mBio.01115-21.1 FIG S1 VgrG1_725_-Cre_3V5_ fusion can deliver TseL to kill sensitive prey. T6SS competition assay showing killing by wild-type V52 (WT) or a V52 strain lacking the *vgrG1* gene (Δ*vgrG1*) and complemented with no plasmid (n/a), VgrG1_725_-Cre_3V5,_ or VgrG3_717_-Cre_3V5_. Prey are a V52 strain lacking the TseL immunity gene, *tsiV1*, and complemented with either empty vector (pVec) or *tsiV1* on a plasmid (pImm). Survival of the prey is shown as a ratio of recovered CFU (pVec/pImm). One-way ANOVA with Dunnett’s multiple-comparison test compared each killer strain to Δ*vgrG1* with no plasmid. **, *P* < 0.01; ***, *P* < 0.001; ns, not significant. Download FIG S1, TIF file, 0.04 MB. Copyright © 2021 Hersch et al. 2021 Hersch et al. https://creativecommons.org/licenses/by/4.0/ This content is distributed under the terms of the Creative Commons Attribution 4.0 International license.

### Active VgrG-Cre fusions are delivered into recipient cells.

We next sought to determine if the engineered fusions could deliver active Cre recombinase into neighboring cells. We constructed a plasmid-based cassette for detecting Cre-mediated recombination in recipient cells. The plasmid contains a floxed (flanked by *loxP* sites) ampicillin resistance (Amp^r^) cassette that interrupts translation of a gentamicin resistance (Gent^r^) gene (Fig. 1C). We termed this plasmid pFIGR, for floxed interruption in gentamicin resistance. Before Cre recombination, pFIGR grants ampicillin resistance but the cells remain gentamicin sensitive. Upon Cre-mediated recombination, the block is lifted and the Gent^r^ gene is expressed, granting gentamicin resistance to the bacteria. This provides a simple method for detecting Cre recombination as growth of recipient cells on media containing gentamicin. Notably, recipients would only become sensitive to ampicillin if all copies of pFIGR (approximately 20/cell) lost the Amp^r^ cassette.

To avoid killing the recipients with T6SS effectors, we used the same V. cholerae V52 background, encoding all immunity genes, as the recipient. Furthermore, to prevent resecretion of the fusions by the T6SS of recipient cells, the Δ*tssM* mutant was used as the recipient strain. Finally, for enumeration of total recipients, we also introduced a kanamycin-resistant (Kan^r^) plasmid that is compatible with pFIGR.

After combining donor cells and recipients containing pFIGR, we found that all four of the VgrG-Cre fusions led to significant levels of Gent^r^ CFU (Fig. 1D). The VgrG3_717_-Cre_3V5_ construct was the most effective, leading to about 500-fold more Gent^r^ CFU than controls. Importantly, we confirmed that the gain of gentamicin resistance required Cre, since controls lacking Cre demonstrated background levels of Gent^r^ CFU. Moreover, three pieces of evidence support that the observed gentamicin resistance was dependent on direct T6SS-mediated delivery: (i) a Cre-only control that lacks a VgrG delivery tag did not increase the number of Gent^r^ colonies. (ii) Wild-type donors demonstrated significantly more Cre delivery (Gent^r^ CFU) than T6SS-null (Δ*tssM*) strains (Fig. 1D, Fig. S2A). (iii) Attempts to deliver Cre under conditions that are prohibitive of the T6SS, including in liquid media or with a barrier separating the donor and recipient cells, failed to increase the yield of Gent^r^ colonies (Fig. S2B and C). Notably, Cre delivery does not appear to recombine every copy of pFIGR in a cell, since Amp^r^ (nonrecombined FIGR) and Kan^r^ (total recipient) CFU counts were indistinguishable, suggesting that some copies of pFIGR retain the Amp^r^ gene (Fig. S2A).

10.1128/mBio.01115-21.2 FIG S2 VgrG fusions total donor and recipient survival, liquid, or barrier controls, related to Fig. 1D. (A) Recovery of V. cholerae Kan^r^ (total recipients) or Carb^r^ (recipients with at least one nonrecombined copy of FIGR) colony-forming units after delivery from wild-type (+) or Δ*tssM* (Δ) V. cholerae with indicated Cre fusions to VgrG vectors. One-way ANOVA with Sidak’s multiple-comparison test comparing wild-type and Δ*tssM* donors with comparable delivery fusions. Kan^r^ statistics are shown, and similar results were obtained for Carb^r^. No significant differences were found between Kan^r^ and Carb^r^ results for any delivery fusion strains. ***, *P* < 0.001; ns, not significant. (B and C) Cre delivery in liquid suspension (liquid) or when donor and recipient were separated by a nitrocellulose membrane (barrier). Recovery of V. cholerae colony-forming units after incubating with wild-type V. cholerae with indicated Cre fusions. One-way ANOVA indicated no significant differences. (B) Gent^r^ CFU numbers indicative of Cre-mediated FIGR cassette recombination. DL, detection limit. (C) Total recipient CFU numbers (Kan^r^). (D) Recovery of V. cholerae Cm^r^ colony-forming units (total donor cell recovery) with indicated Cre fusions to VgrG vectors after incubation with V. cholerae FIGR recipients. One-way ANOVA with Dunnett’s multiple-comparison test compared each strain to the Cre only strain. *, *P* < 0.05; **, *P* < 0.01; ***, *P* < 0.001; ns, not significant. Download FIG S2, TIF file, 0.3 MB. Copyright © 2021 Hersch et al. 2021 Hersch et al. https://creativecommons.org/licenses/by/4.0/ This content is distributed under the terms of the Creative Commons Attribution 4.0 International license.

Since the T6SS has been associated with horizontal gene transfer, we also considered that transfer of either pFIGR or the Cre donor plasmid (which is chloramphenicol resistant; Cm^r^) could result in a strain containing both plasmids, leading to Gent^r^ colonies. However, this did not appear to occur, since the Cre-only control plasmid did not increase the numbers of Gent^r^ CFU (Fig. 1D) and not a single Cm^r^ Gent^r^ double-resistant colony was detected across all replicates (data not shown). These data suggest that the observed Gent^r^ colonies resulted from genuine T6SS-mediated delivery of active Cre protein.

Data from this initial Cre delivery experiment revealed some limitations to be addressed. First, the VgrG-Cre fusions appear to restrict the donor cells, leading to reduced recovery compared to no-Cre or Cre-only controls (Fig. S2D). This was independent of T6SS activity, suggesting that the VgrG-Cre fusions result in undetermined toxicity or exert a resource strain on donor cells expressing the large recombinant proteins. The second limitation was that we observed reduced recipient survival with wild-type donors compared to the Δ*tssM* strain (Fig. S2A), suggesting that T6SS effectors were killing recipient cells despite the recipient strains encoding all immunity genes. This was particularly evident when delivering VgrG3 fusions; it also occurred with strains expressing Cre only (no delivery fusion) or VgrG1/3-nc (no Cre), suggesting that Cre itself was not instigating the toxicity (Fig. S2A). These limitations are addressed in the following sections.

### Using a PAAR2-Cre delivery vector improves donor fitness.

**FIG 2:**
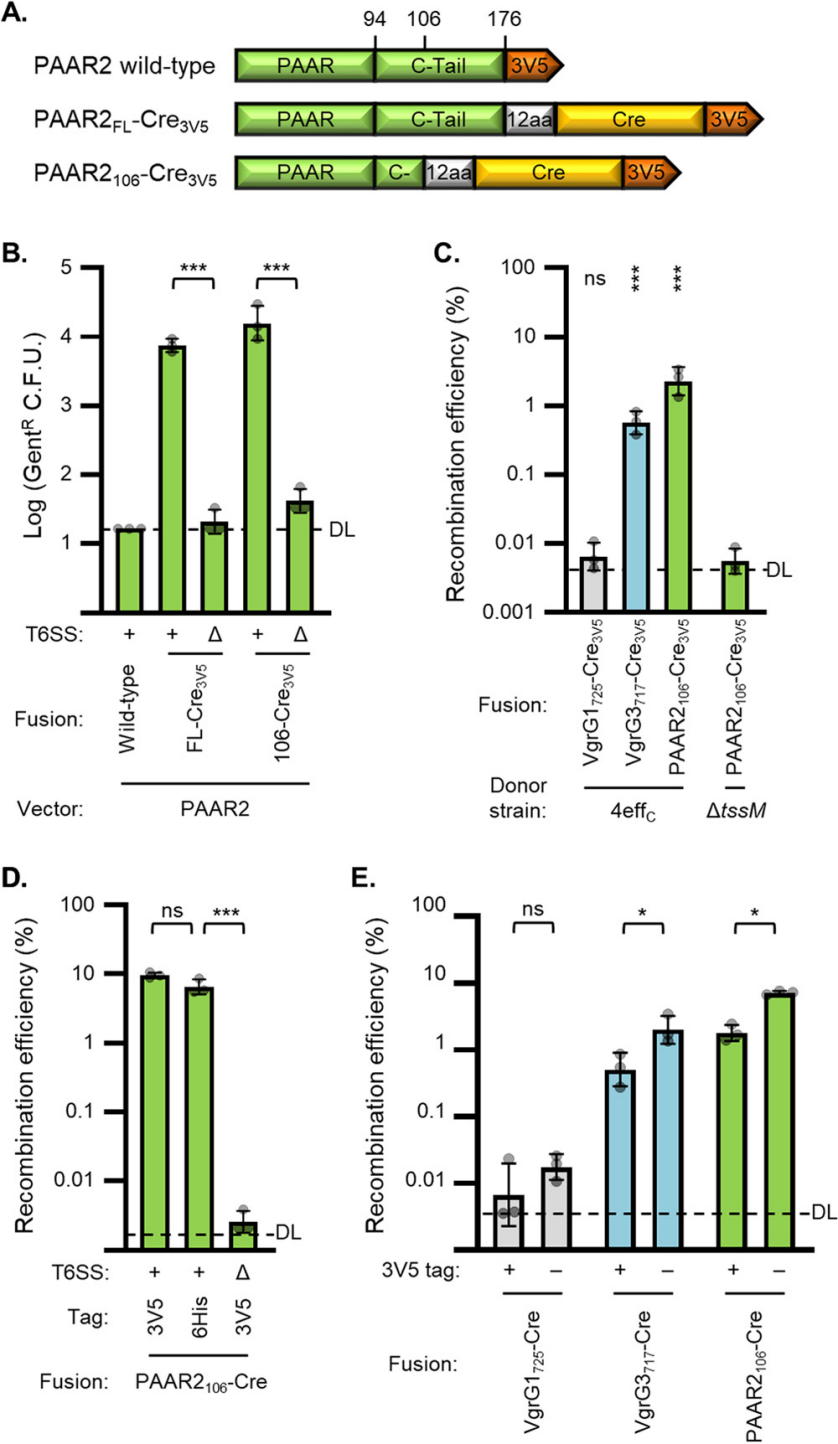
Improving Cre delivery using PAAR2 fusions, an effectorless donor strain, and removing 3V5 tags. (A) Depiction (not to scale) showing fusions of Cre recombinase (yellow) and 3× V5 tag (orange) to PAAR2 (green) of V. cholerae V52. Fusions include the full-length C-terminal tail (C-Tail) of PAAR2 (FL) or just 12 amino acids of it (C-); 12 amino acids (12aa) are also left over from the VgrG1_725_-Cre_3V5_ construct (codons 714 to 725), used as the template for PAAR2 insertion. (B) Recovery of Cre-recombined recipient bacteria (Gent^r^ CFU) after delivery from wild-type (+) or Δ*tssM* (Δ) V. cholerae with indicated Cre fusions to PAAR2 vectors. DL, detection limit. One-way ANOVA with Sidak’s multiple-comparison test. Recovery from Δ*tssM* samples was not significantly above the no-Cre (PAAR2 wild-type) control. ***, *P* < 0.001. (C) Cre fusion delivery from V. cholerae with catalytically inactivated antibacterial effectors (4eff_C_) or Δ*tssM* (Δ). Data show recombination efficiency (Gent^r^/Kan^r^ CFU recovered; recombined/total recipients) as a percentage. DL, approximate detection limit. One-way ANOVA with Dunnett’s multiple-comparison test comparing each sample to the Δ*tssM* donor strain. ***, *P* < 0.001; ns, not significant. (D) Recombination efficiency after delivery from 4eff_C_ (+) or Δ*tssM* (Δ) V. cholerae encoding PAAR_106_-Cre with either a 3V5 or 6His tag. DL, approximate detection limit. One-way ANOVA with Tukey’s multiple-comparison test. ***, *P* < 0.001; ns, not significant. (E) Recombination efficiency after delivery from 4eff_C_ strain encoding indicated Cre fusions with the 3V5 tag present (+) or removed (−). DL, approximate detection limit. One-way ANOVA with Sidak’s multiple-comparison test comparing samples with and without 3V5 tags. *, *P* < 0.05; ns, not significant.

Since expressing the VgrG-Cre fusions conveyed a fitness cost on the donor cells (Fig. S2D; as described above), we sought an alternative delivery fusion that would not inhibit the donor cells. PAAR2 in V. cholerae does not have an effector domain, but, in addition to its N-terminal PAAR domain, it does include a C-terminal tail for recruiting the effector, TseH (17). Full-length PAAR2 is only 176 amino acids long, which is a significantly smaller T6S tag than the VgrG fusions; we hypothesized that the smaller size would reduce the metabolic burden of expressing it. We fused Cre to either full-length PAAR2 (PAAR2_FL_) or a truncated version (PAAR2_106_) that has only 12 amino acids of the C-terminal tail (Fig. 2A). Notably, since the VgrG1-Cre plasmid was used as a template, the cloning method left a 12-amino-acid remnant of VgrG1 (codons 714 to 725, downstream of polyproline region, sequence TANAQPNLGRST); these residues act as a further linker between PAAR2 and Cre.

Delivery of either PAAR2_FL_-Cre_3V5_ or PAAR2_106_-Cre_3V5_ led to significant and substantial Cre-mediated recombination in recipient cells (Fig. 2B). The number of Gent^r^ recipients following PAAR2_106_-Cre_3V5_ delivery was slightly higher than that for PAAR2_FL_-Cre_3V5_ and comparable to the most effective fusion tested previously, VgrG3_717_-Cre_3V5_ (Fig. 2B compared to 1D). The no-Cre control (wild-type PAAR2) and all Δ*tssM* donors yielded undetectable or background amounts of recombination, demonstrating that recombination in recipients required T6SS-mediated secretion of Cre. As expected, delivery of the PAAR2-Cre fusions was also contact dependent, since delivery in liquid media or with a barrier separating the donor and recipient cells yielded negligible Gent^r^ CFU numbers (Fig. S3A and B). Importantly, expression of the PAAR2-Cre fusions did not reduce donor cell fitness (Fig. S3C), suggesting that they did not cause a fitness cost like the VgrG-Cre fusions. However, delivery of these fusions from wild-type donor cells still demonstrated some toxicity to the recipients (Fig. S3D).

10.1128/mBio.01115-21.3 FIG S3 PAAR2 fusions total donor and recipient survival, liquid, or barrier controls, related to Fig. 2B. (A and B) Cre delivery in liquid suspension (liquid) or when donor and recipient were separated by a nitrocellulose membrane (barrier). Recovery of recipient colony forming units after incubating with wild-type V. cholerae with PAAR2_106_-Cre_3V5_. (A) Gent^r^ CFU indicative of Cre-mediated FIGR cassette recombination. DL, detection limit. (B) Total recipient CFU numbers (Kan^r^). (C) Recovery of V. cholerae Cm^r^ colony-forming units (total donor cell recovery) with indicated Cre fusions to PAAR2 after incubation with V. cholerae FIGR recipients. One-way ANOVA analysis indicates no significant differences. (D) Recovery of V. cholerae Kan^r^ (total recipients) colony-forming units after delivery from active T6SS (+) or Δ*tssM* (Δ) V. cholerae with indicated Cre fusions to PAAR2 vectors. One-way ANOVA with Sidak’s multiple-comparison test comparing active T6SS and Δ*tssM* donors with comparable delivery fusions. **, *P* < 0.01; ***, *P* < 0.001. Download FIG S3, TIF file, 0.1 MB. Copyright © 2021 Hersch et al. 2021 Hersch et al. https://creativecommons.org/licenses/by/4.0/ This content is distributed under the terms of the Creative Commons Attribution 4.0 International license.

### Inactivating donor effectors restores recipient survival.

Despite using V. cholerae recipients, with all immunity genes intact, we consistently observed some degree of toxicity toward the recipients that was independent of which Cre fusion was being delivered (Fig. S2A and S3D). To address this limitation, we switched donor strains to a previously generated V52 strain that has all four of its antibacterial effectors catalytically inactivated (4eff_C_) (16, 17). The 4eff_C_ strain showed no toxicity to recipients (Fig. S4A). Notably, delivery of VgrG-Cre fusions was reduced when delivered from the 4eff_C_ strain compared to the wild-type donor (Fig. S4B compared to Fig. 1D). However, delivery of the PAAR2_106_-Cre_3V5_ fusion from the 4eff_C_ donor strain was similar to that from the wild type (Fig. S4B compared to Fig. 2B). These data support using the 4eff_C_ strain to solve the recipient toxicity issue.

10.1128/mBio.01115-21.4 FIG S4 Cre fusion delivery from effectorless donor strain, related to Fig. 2C. (A) Total recipients recovered (Kan^r^ CFU) after delivery from V. cholerae with catalytically inactivated antibacterial effectors (4eff_C_) or Δ*tssM* (Δ). One-way ANOVA with Dunnett’s multiple-comparison test comparing each sample to the Δ*tssM* donor strain. ***, *P* < 0.001; ns, not significant. (B) As for panel A but showing Cre-recombined recipient bacteria (Gent^r^ CFU). DL, detection limit. One-way ANOVA indicates no significant differences. Download FIG S4, TIF file, 0.1 MB. Copyright © 2021 Hersch et al. 2021 Hersch et al. https://creativecommons.org/licenses/by/4.0/ This content is distributed under the terms of the Creative Commons Attribution 4.0 International license.

### Measuring recombination efficiency and further improving efficacy.

Using the 4eff_C_ donor strain, toxicity was prevented and total recipient recovery was consistent regardless of which fusion construct was delivered (Fig. S4A). This allowed for measuring recombination efficiency by dividing the number of recombined recipients (Gent^r^ CFU) by the number of total recipients (Kan^r^ CFU). Using this metric, we observed up to about 2.5% recombination efficiency using the PAAR2_106_-Cre_3V5_ construct (Fig. 2C).

We considered that the C-terminal 3× V5 tag might inhibit delivery or Cre activity; we experimented using a 6× His tag (6×His) or removing the detection tag entirely. The 6×His tag did not reduce recombination efficiency (Fig. 2D), suggesting that 6×His-tagged proteins can be successfully secreted using the PAAR2_106_ vector. Removal of the detection tag significantly improved recombination efficiency for both VgrG3_717_- and PAAR2_106_-Cre fusions (Fig. 2E), raising the efficiency to over 7%. Oddly, in the 6×His experiment (Fig. 2D), the PAAR2_106_-Cre_3V5_ construct exhibited up to 10% recombination efficiency, highlighting that there is some variability between experiments.

### T6SS effectors can serve as delivery vectors.

**FIG 3:**
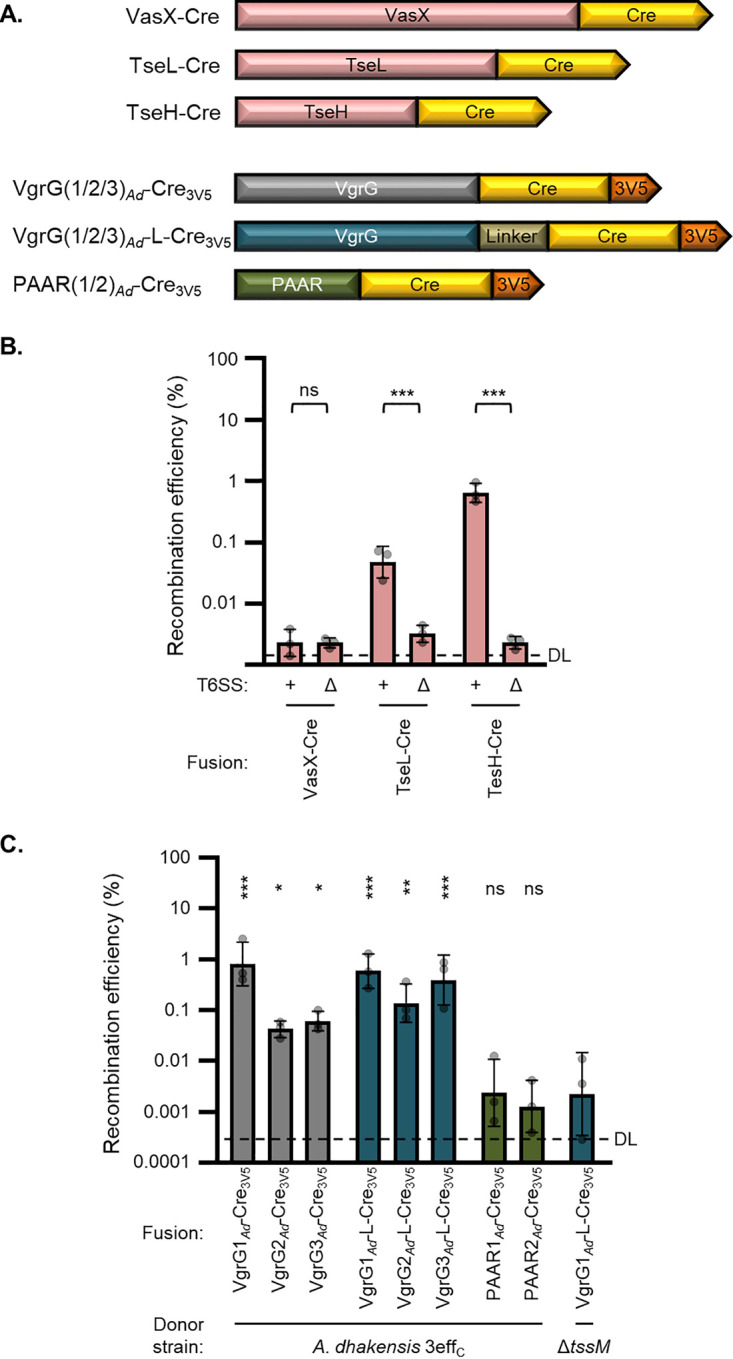
Active Cre can be delivered as effector fusions and by *A. dhakensis*. (A) Depiction (not to scale) showing fusions of Cre recombinase (yellow) and 3× V5 tag (orange) to V. cholerae T6SS effectors (red) or to *A. dhakensis* PAAR proteins (dark green) and VgrG proteins with (dark blue) or without (dark gray) a flexible linker inserted. (B) Recombination efficiency after delivery from V. cholerae 4eff_C_ (+) or Δ*tssM* (Δ) strains encoding Cre fusions to indicated effector proteins. DL, approximate detection limit. One-way ANOVA with Sidak’s multiple-comparison test comparing 4eff_C_ and Δ*tssM* donors with equivalent delivery fusions. ***, *P* < 0.001; ns, not significant. (C) Recombination efficiency after delivery of Cre fusions from *A. dhakensis* with catalytically inactivated antibacterial effectors (3eff_C_) or Δ*tssM* (Δ). DL, approximate detection limit. One-way ANOVA with Dunnett’s multiple-comparison test comparing each sample to the Δ*tssM* donor. *, *P* < 0.05; **, *P* < 0.01; ***, *P* < 0.001; ns, not significant.

Up to this point, we have demonstrated delivery of Cre as a fusion to T6SS structural proteins, VgrG1, VgrG3, and PAAR2 of V. cholerae. To further determine the versatility of T6SS-mediated protein delivery, we fused Cre to the C termini of the three V. cholerae cargo effectors, VasX, TseL, and TseH (Fig. 3A). The VasX-Cre construct did not facilitate recombination in recipient cells (Fig. 3B) or kill VasX-sensitive prey (Fig. S5), suggesting that this fusion was inactive or failed to be secreted. However, both TseL and TseH fusions demonstrated significant Cre delivery (Fig. 3B). The efficiency of TseH-Cre delivery rivalled VgrG3_717_-Cre_3V5_ but was lower than that for PAAR2_106_ fusions (Fig. 3B compared to 2E). These findings demonstrate that nonstructural proteins can also serve as T6SS delivery tags to deliver customized cargo proteins.

10.1128/mBio.01115-21.5 FIG S5 VasX-Cre fusion does not kill sensitive prey. T6SS competition assay showing killing by V52 wild-type (WT), catalytically inactivated antibacterial effectors (4eff_C_), or inactivated T6SS (Δ*tssM*). Strains were complemented with no plasmid (n/a) or VasX-Cre. Prey are a V52 strain lacking the VasX immunity gene, *tsiV2*, and complemented with either *tsiV2* (pImm) or a nonprotective chaperone gene as a control (VCA0019; pCtrl). Survival of the prey is shown as a ratio of recovered CFU numbers (pCtrl/pImm). One-way ANOVA with Sidak’s multiple-comparison test comparing no plasmid WT or VasX-Cre delivery to controls. ***, *P* < 0.001; ns, not significant. Download FIG S5, TIF file, 0.04 MB. Copyright © 2021 Hersch et al. 2021 Hersch et al. https://creativecommons.org/licenses/by/4.0/ This content is distributed under the terms of the Creative Commons Attribution 4.0 International license.

### *A. dhakensis* is another proficient donor strain.

We were curious if other species could also be employed for T6SS-mediated delivery of recombinant proteins. We tested another aquatic species that has a constitutively active T6SS, *A. dhakensis* strain SSU (30, 34). We fused Cre to each of the three VgrGs and two PAAR proteins present in *A. dhakensis* (Fig. 3A). We then introduced these constructs into an *A. dhakensis* strain with its three antibacterial effectors, TseI, TseP, and TseC, all catalytically inactivated (3eff_C_) (35). This strain showed no toxicity toward V. cholerae recipients (Fig. S6B) (35).

10.1128/mBio.01115-21.6 FIG S6 *A. dhakensis* donor total recipient survival and recombinant recovery, related to Fig. 3C. (A) Recovery of Cre-recombined recipient bacteria (Gent^r^ CFU) after delivery of Cre fusions from *A. dhakensis* with catalytically inactivated antibacterial effectors (3eff_C_) or Δ*tssM* (Δ). DL, detection limit. One-way ANOVA with Dunnett’s multiple-comparison test comparing each sample to the Δ*tssM* donor. **, *P* < 0.01; ***, *P* < 0.001; ns, not significant. (B) As for panel A but showing total recipients recovered (Kan^r^ CFU). One-way ANOVA analysis indicates no significant differences. (C) Recovery of *A. dhakensis* Cm^r^ colony-forming units (total donor cell recovery) with indicated Cre fusions after incubation with V. cholerae FIGR recipients. One-way ANOVA with Dunnett’s multiple-comparison test compared each sample to the Δ*tssM* donor. **, *P* < 0.01; ns, not significant. Download FIG S6, TIF file, 0.2 MB. Copyright © 2021 Hersch et al. 2021 Hersch et al. https://creativecommons.org/licenses/by/4.0/ This content is distributed under the terms of the Creative Commons Attribution 4.0 International license.

All three VgrG fusions led to significant Cre delivery from the T6SS^+^ donor compared to an equivalent Δ*tssM* strain (Fig. 3C); delivery of VgrG1*_Ad_*-Cre_3V5_ yielded the highest recombination efficiency. In contrast, neither of the *A. dhakensis* PAAR fusions demonstrated signs of delivery. Since none of the *A. dhakensis* VgrG proteins have effector domains, we attempted to better mimic an evolved VgrG by introducing a flexible linker (sequence SGGGSGGGSGGG) between Cre and the VgrG domain (Fig. 3A). The linker appeared to improve delivery for VgrG2*_Ad_* and VgrG3*_Ad_* but not VgrG1*_Ad_* (Fig. 3C). Notably, none of the fusion constructs appeared to inhibit donor fitness, and the 3eff_C_ strain showed no recipient toxicity compared to the Δ*tssM* strain (Fig. S6). Cumulatively, these findings demonstrate that the T6SS of multiple species can be engineered to deliver active Cre recombinase. Additionally, this transfer can cross species barriers (*A. dhakensis* delivery to V. cholerae recipients) if the donor’s antibacterial effectors are inactivated.

We also examined delivery to a recipient strain with a chromosomal copy of FIGR instead of the pFIGR plasmid (approximately 20 copies per cell). Delivery of the VgrG3_Ad_-L-Cre_3V5_ fusion from *A. dhakensis* resulted in slight but significant levels of recombination efficiency (Fig. S7). This suggests that chromosomal FIGR can detect Cre-mediated recombination to a measurable level, though it appears to be less efficient than the plasmid-borne cassette.

10.1128/mBio.01115-21.7 FIG S7 Cre delivery to V. cholerae recipients with chromosomal FIGR. (A) Recombination efficiency of V. cholerae recipients with chromosomal FIGR cassette after delivery from V. cholerae 4eff_C_ (+) or Δ*tssM* (Δ) strains. (B) Recombination efficiency of V. cholerae recipients with chromosomal FIGR cassette after delivery from *A. dhakensis 3*eff_C_ (+) or Δ*tssM* (Δ) strains. DL, approximate detection limit. For each experiment, one-way ANOVAs with Dunnett’s multiple-comparison tests compared samples to Δ*tssM* donors. *, *P* < 0.05; ns, not significant. Download FIG S7, TIF file, 0.1 MB. Copyright © 2021 Hersch et al. 2021 Hersch et al. https://creativecommons.org/licenses/by/4.0/ This content is distributed under the terms of the Creative Commons Attribution 4.0 International license.

### The PAAR2_106_ T6S tag enables V. cholerae to deliver a recombinant effector to kill P. aeruginosa.

To examine if engineered T6S tags can deliver proteins other than Cre, we focused on delivering exogenous T6SS effectors. Customizing effector delivery could be used to study effector activities or to maximize efficacy against particular prey. As a proof of principle, we employed V. cholerae and P. aeruginosa. It was previously shown that the T6SS of V. cholerae does not kill P. aeruginosa (27); however, the mechanism remains unclear. One possibility is that V. cholerae effectors are not active against P. aeruginosa; alternatively, the V. cholerae T6SS might not be able to puncture the P. aeruginosa cell envelope to deliver the effectors (17, 27, 36).

**FIG 4:**
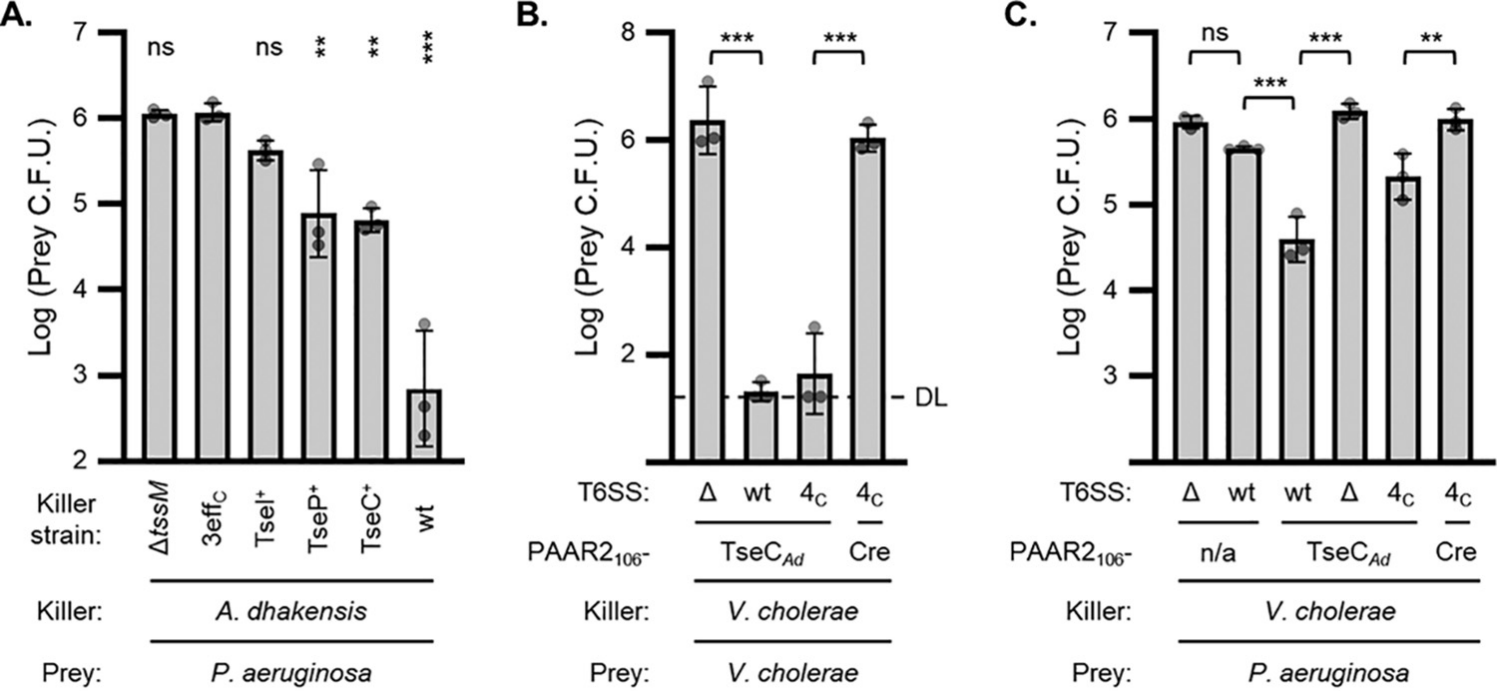
Fusion of PAAR2_106_ to the *A. dhakensis* effector, TseC, empowers V. cholerae to kill P. aeruginosa. (A) Prey P. aeruginosa recovery after incubation with *A. dhakensis* killer cells. Prey P. aeruginosa has Δ*hsiB* (Δ*tssB*) mutations in all three T6SS. Killer strains have T6SS-null mutation (Δ*tssM*) or a wild-type T6SS with all effectors intact (wt), all antibacterial effectors catalytically inactivated (3eff_C_), or a single active effector (indicated), while the other effectors are inactivated. One-way ANOVA with Dunnett’s multiple-comparison test compared all samples to the 3eff_C_ killer strain. **, *P* < 0.01; ***, *P* < 0.001; ns, not significant. (B) Prey V. cholerae (Δ*tssM*) recovery after incubation with V. cholerae killer cells encoding PAAR2_106_ fusions to *A. dhakensis* TseC (TseC*_Ad_*) or Cre as a control. Killer strains have Δ*tssM* (Δ) or a wild-type T6SS with all effectors intact (wt) or all native antibacterial effectors catalytically inactivated (4eff_C_). DL, detection limit. One-way ANOVA with Sidak’s multiple-comparison test. ***, *P* < 0.001; ns, not significant. (C) Prey P. aeruginosa (Δ*hsiB* mutations in all three T6SS) recovery after incubation with V. cholerae killer cells with no fusion construct (n/a) or encoding PAAR2_106_ fusions to *A. dhakensis* TseC (TseC*_Ad_*) or Cre as a control. Killer strains have Δ*tssM* (Δ) or a wild-type T6SS with all effectors intact (wt) or all native antibacterial effectors catalytically inactivated (4eff_C_). One-way ANOVA with Sidak’s multiple-comparison test. **, *P* < 0.01; ***, *P* < 0.001; ns, not significant.

We employed T6S fusions to elucidate this mystery. We noticed that *A. dhakensis* can use its T6SS to kill P. aeruginosa, and this lethality is abolished when the three antibacterial effectors are catalytically inactivating (Fig. 4A). By inactivating two effectors at a time, leaving a single active effector in the strain, we determined that TseP and TseC were effective against P. aeruginosa and TseC was more consistent (Fig. 4A). In light of this, we replaced Cre (of the PAAR2_106_-Cre fusion) with TseC and its downstream immunity gene (*tsiC*) from *A. dhakensis*. Both the wild-type and 4eff_C_ strains of V. cholerae were able to deliver this PAAR2_106_-TseC*_Ad_* fusion, resulting in greatly reduced survival of competing V. cholerae (Fig. 4B). Moreover, expressing PAAR2_106_-TseC*_Ad_* enabled both wild-type and 4eff_C_ V. cholerae to kill P. aeruginosa (Fig. 4C). Importantly, this killing was T6SS and TseC*_Ad_* dependent (Fig. 4C), and expression of the immunity gene, *tsiC*, in prey cells was protective (Fig. S8). Cumulatively, these data suggest that reduced P. aeruginosa survival results from T6SS-mediated delivery of the *A. dhakensis* effector from V. cholerae. Accordingly, V. cholerae delivering PAAR2_106_-TseC*_Ad_* inhibited P. aeruginosa to a similar degree as the *A. dhakensis* strain with TseC as its only active effector (Fig. 4A compared to C).

10.1128/mBio.01115-21.8 FIG S8 Immunity gene, *tsiC*, protects against TseC and PAAR2_106_-TseC*_Ad_*. (A) Recovery of prey P. aeruginosa with empty vector (pVec) or expressing TsiC (pTsiC) after incubation with *A. dhakensis* killer cells. Prey P. aeruginosa has Δ*hsiB* (Δ*tssB*) mutations in all three T6SS. Killer strains have all antibacterial effectors catalytically inactivated (3eff_C_), or all effectors are inactivated except for TseC (TseC^+^). One-way ANOVA with Sidak’s multiple-comparison test comparing corresponding pVec and pTsiC samples. ***, *P* < 0.001; ns, not significant. (B) Recovery of prey V. cholerae (Δ*tssM*) with vector (pVec) or expressing TsiC (pTsiC) after incubation with V. cholerae killer cells expressing the PAAR2_106_-TseC*_Ad_* fusion. Killer strains have Δ*tssM* (Δ) or a wild-type T6SS with all effectors intact (wt) or all native antibacterial effectors catalytically inactivated (4eff_C_). DL, detection limit. One-way ANOVA with Sidak’s multiple-comparison test comparing corresponding pVec and pTsiC samples. ***, *P* < 0.001; ns, not significant. (C) Recovery of prey P. aeruginosa (Δ*hsiB* mutations in all three T6SS) with vector (pVec) or expressing TsiC (pTsiC) after incubation with V. cholerae killer cells expressing the PAAR2_106_-TseC*_Ad_* fusion. Killer strains have Δ*tssM* (Δ) or a wild-type T6SS with all effectors intact (wt) or all native antibacterial effectors catalytically inactivated (4eff_C_). One-way ANOVA with Sidak’s multiple-comparison test comparing corresponding pVec and pTsiC samples. ***, *P* < 0.001; ns, not significant. Download FIG S8, TIF file, 0.1 MB. Copyright © 2021 Hersch et al. 2021 Hersch et al. https://creativecommons.org/licenses/by/4.0/ This content is distributed under the terms of the Creative Commons Attribution 4.0 International license.

## DISCUSSION

**FIG 5:**
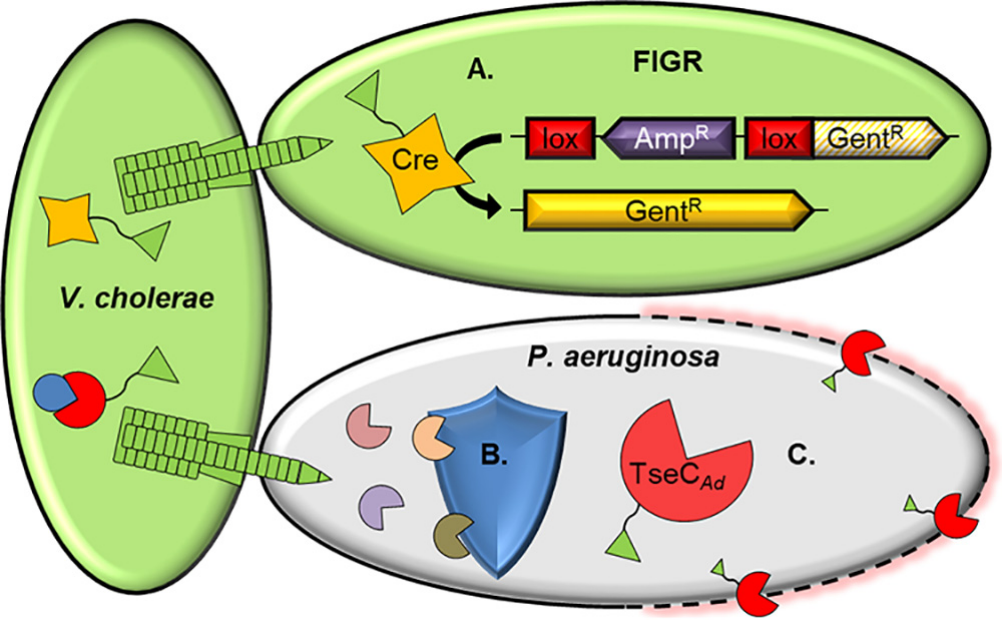
Model of engineered T6SS-mediated delivery of Cre recombinase and TseC*_Ad_*. (A) Engineered T6SSs of V. cholerae or *A. dhakensis* donor cells deliver active Cre recombinase protein. Delivered Cre instigates recombination of the floxed interruption in the gentamicin resistance (FIGR) cassette in recipient cells. (B) P. aeruginosa exhibits natural resistance (by unknown mechanisms) to all four antibacterial effectors of wild-type V. cholerae. (C) V. cholerae engineered to deliver the *A. dhakensis* effector, TseC*_Ad_*, gains the ability to kill P. aeruginosa.

In this work, we developed multiple fusion tags allowing the T6SSs of V. cholerae and *A. dhakensis* to efficiently deliver the targeted endonuclease, Cre, or an exogenous effector, TseC*_Ad_*, directly into neighboring cells (Fig. 5). We also showed activity of the system with a 6×His-tagged protein, potentially leading to a downstream method for lysis-free purification of secreted fusion proteins. Additionally, we constructed a novel gain-of-function Cre detection cassette (FIGR) and identified the *A. dhakensis* T6SS effectors that are most active against P. aeruginosa.

The delivery fusion results described in this work reveal details of T6SS function. For instance, since Cre was able to access the DNA of recipient cells, it implies that the T6SS tip can puncture into the cytoplasm. This supports previous works using nuclease effectors, the reuse of secreted T6SS components in recipient sister cells, and the finding that VgrG3 of V. cholerae has evolved to reexport from the cytoplasm of recipient cells to access its peptidoglycan target in the periplasm (22, 37). Another finding was that Cre could be delivered as a fusion to PAAR2 of V. cholerae but not when fused to either PAAR protein of *A. dhakensis*. Likely, this results from the extended C-terminal domain of PAAR2 that is not present in the other PAAR proteins. This region acts as a linker (even when truncated in the PAAR2_106_ construct) that potentially improves the folding or functionality of Cre or the PAAR domain. This suggests that effectors that are attached to PAAR domains must include a sufficient linker or else impede folding or functionality.

We employed the PAAR2_106_ T6S tag to engineer V. cholerae to deliver the *A. dhakensis* effector, TseC. This led to a number of significant findings. (i) TseC was fully active when delivered from V. cholerae, suggesting that TseC’s chaperone (TEC) protein is not required for proper folding or activity (30). However, V. cholerae also encodes two DUF4123 TEC proteins, so it remains possible that they can act in lieu of the *A. dhakensis* chaperone (30). (ii) Since the *tseC* fusion gene demonstrated full activity after switching to a new host organism and delivery mechanism, this provides further empirical evidence that domain swapping can facilitate new effector integration. This supports previous work examining the evolution of the T6SS by horizontal gene transfer and the modular nature of effectors (22, 38–41).

The findings additionally highlight the evolutionary advantage of accumulating exogenous effectors, since V. cholerae equipped with PAAR2_106_-TseC devastated neighboring V. cholerae populations (Fig. 4B). Moreover, it gained the ability to kill P. aeruginosa, which suggests that the V. cholerae T6SS is capable of delivering effectors into P. aeruginosa, but these prey cells have the ability to resist killing by all four of V. cholerae’s natural antibacterial T6SS effectors. This resistance may not be complete, as delivery of V. cholerae effectors can instigate a retaliatory tit-for-tat response in P. aeruginosa (17, 27). Additionally, delivering PAAR2_106_-TseC*_Ad_* from the wild-type donor appeared to kill P. aeruginosa more than delivery from the 4eff_C_ strain, suggesting that there is some level of synergy between TseC and the V. cholerae effectors. Nonetheless, the V. cholerae effectors alone did not significantly reduce P. aeruginosa survival. The mechanism of this resistance remains to be determined in future work but could involve cross-protection by P. aeruginosa’s array of immunity genes or alternative tolerance systems, such as stress response-mediated damage repair. This finding further supports a growing body of work highlighting that certain effectors are more efficacious against particular prey cells or under particular conditions (16, 17, 42–44).

The antibacterial activity of the T6SS can be harnessed to target specific species for manipulating microbiomes or as a potential next-generation antimicrobial (21, 22, 45). To achieve this, it is crucial to be able to equip the T6SS with a customized selection of effectors. Effector discovery used to be a major challenge of the field due to sequence divergence, but multiple approaches have been developed to overcome this earlier challenge (23, 30, 46–48). Now the pool of divergent effectors comprises thousands of proteins in hundreds of species, including many that are genetically intractable. Thus, the ability to swap an effector into a different T6SS delivery species will allow for further study of effectors, and defences against them, in isolation from their native delivery strains. The fusions described here establish delivery mechanisms for auxiliary effectors to maximize efficacy against particular target species, thereby enhancing the potential of the T6SS for future applications.

Finally, the engineered T6SS provides advantages over existing platforms for delivery of genetic editing enzymes as proteins. (i) The T6SS can deliver directly into the target cell cytoplasm without requiring a receptor. This grants an advantage over secreted proteins, which require receptors for uptake, deliver only to the periplasm of bacteria, or both. (ii) The T6SS can deliver ready-folded proteins, providing an advantage over T3SSs or T4SSs for cargo that is inactivated by denaturation. (iii) Due to its receptor-independent mechanism, the T6SS has a broad spectrum of target cells, including both eukaryotic and prokaryotic cells. The system described here provides the first example of delivering a genetic editing enzyme as a protein into bacteria.

Throughout the process of optimizing the T6SS delivery fusions, we employed Cre recombinase. Notably, Cre acts as a tetramer, requiring at least four molecules to be delivered to recipients before recombination can occur. Since three VgrG proteins are delivered per firing event, the VgrG-Cre fusions potentially deliver multiple Cre molecules at once. However, it is not known if the VgrG trimer dissociates in recipient cells or if Cre instigates recombination while still assembled in a VgrG trimer. In contrast, the PAAR2_106_ fusion tag can only deliver one protein per T6SS firing event but may promote Cre functionality due to the smaller T6S tag domain. Because it functions as a tetramer, Cre recombination likely understates delivery efficiency, which may increase with the use of monomeric enzymes in the future.

In addition to the immediate potential applications of Cre delivery, it primarily acts as a proof of principle. The successful delivery of Cre, and subsequent recombination in recipient cells, marks an important landmark for the use of the T6SS in genetic engineering methods. Vector integration remains a significant constraint in genetic editing, but protein delivery using the T6SS can potentially eliminate this issue. Dosage can also be readily regulated to limit off-target effects by controlling donor/recipient ratios, repressing T6S tag expression, or using antibiotics to rapidly end endonuclease delivery by killing the donor bacteria. Future work, including with additional gene editing tools such as CRISPR-Cas9, will further develop this protein delivery system.

## MATERIALS AND METHODS

### Bacterial strains and growth conditions.

Strains used in this study are listed in Table S1A in the supplemental material. All V. cholerae strains were from the V52 strain background, with deletions of *rtxA*, *hlyA*, and *hapA* (RHH) (9). Unless otherwise indicated, recipients were V. cholerae Δ*tssM* with pFIGR and pBAD33k as an independent, compatible plasmid for total CFU enumeration. P. aeruginosa prey were strain PAO1 organisms with deletion of all three *tssB* (*hsiB*) genes to prevent T6SS retaliation (49) and included the pPSV37 plasmid for selection. Bacteria were grown shaking at 37°C in LB medium (0.5% NaCl) or on LB agar plates. For plasmid maintenance and selection of recipient cells for CFU counts, antibiotics were added to final concentrations of 2.5 μg/ml chloramphenicol, 100 μg/ml carbenicillin, 50 μg/ml kanamycin, and 20 μg/ml gentamicin.

10.1128/mBio.01115-21.9 TABLE S1 Strains and plasmids used in this study. Download Table S1, XLSX file, 0.03 MB. Copyright © 2021 Hersch et al. 2021 Hersch et al. https://creativecommons.org/licenses/by/4.0/ This content is distributed under the terms of the Creative Commons Attribution 4.0 International license.

### Plasmid construction.

Plasmids used in this study are listed in Table S1B. Plasmids were generated using Gibson cloning (50) or overlapping PCR mutagenesis (51) and verified by Sanger sequencing. In brief, pFIGR was generated by first inserting *loxP* sites on both sides of the ampicillin resistance cassette of pBAD24. This floxed Amp^r^ gene was then inserted (in the reverse direction) before the second codon of the gentamicin resistance gene in pPSV37. For chromosomal FIGR, a version of FIGR with Kan^r^ instead of Amp^r^ (FIGR_kan_) was transferred into the pGP-Tn7 plasmid, allowing for transposon-mediated insertion (with the pSTNSK helper plasmid) at the chromosomal *attTn7* site (52).

Delivery fusions were generated in the pBAD33 plasmid backbone (arabinose inducible, chloramphenicol resistant) with a C-terminal 3× V5 tag. Truncated VgrG proteins lacking effector domains were amplified from genomic DNA (gDNA) by PCR and inserted into the vector, followed by insertion of Cre recombinase at sites indicated in Fig. 1A. PAAR2-Cre fusions were generated by replacing VgrG1 with PAAR2_FL_ or PAAR2_106_, leaving a 12-amino-acid remnant of VgrG1 as a linker between PAAR2 and Cre. Subsequent effector, *A. dhakensis*, and TseC fusions were generated by Gibson cloning to replace VgrG or Cre in existing plasmids.

### Secretion assay and Western blotting.

Secretion assays were conducted as described previously (16). In brief, strains were grown for 3 h with arabinose added to 0.4% to induce fusion gene expression. Cultures were centrifuged and pellets stored for sonication as cell lysate samples. Meanwhile, supernatants were passed through 0.22-μm filters. Trichloroacetic acid (TCA) solution was added to a final concentration of 20% and placed at –20°C overnight. Supernatants were then centrifuged at 15,000 × *g* for 20 min at 4°C, pellets were washed with 100% acetone, and the resultant pellet was resuspended in SDS-loading dye for analysis by Western blotting.

As described previously (15, 53), proteins were resolved by polyacrylamide gel electrophoresis, transferred to a nitrocellulose membrane, blocked with 5% skim milk in TBST buffer (50 mM Tris, 150 mM NaCl, and 0.05% Tween 20, pH 7.6) for 1 h at room temperature, and then incubated with primary antibodies overnight at 4°C. Blots were washed 3 times in TBST, incubated with anti-mouse horseradish peroxidase (HRP)-conjugated secondary antibody (Cell Signaling Technology) for 1 h, and then detected using ECL solution (Bio-Rad). Monoclonal antibodies to the V5 epitope tag and RpoB, the beta subunit of RNA polymerase used as a cytoplasmic control, were purchased from GeneTex and NeoClone, respectively.

### T6SS competition and delivery assays.

T6SS activity assays were conducted as described previously, with minor modifications (17). Donor or killer strains were subcultured in LB (with antibiotic for plasmid maintenance as needed) for 3 h with arabinose added to 0.4% (0.01% for *A. dhakensis*) for the last hour. Recipient or prey strains were from overnight cultures. For wild-type Cre donors and equivalent controls, donor and recipient were mixed at a 5:1 ratio. Competition assays and donor experiments with V. cholerae 4eff_C_ (or *A. dhakensis* 3eff_C_), donors employed 10:1 ratios. Mixtures were spotted on LB plates containing 0.1% arabinose (0.01% for *A. dhakensis* donor strains) and incubated for 3 h at 37°C. For pVec/pTsiC induction in Fig. S8, 1 mM isopropyl-β-d-thiogalactopyranoside (IPTG) was also included in the LB plates. Agar plugs containing the mixed bacteria were removed using wide-bore pipette tips, resuspended in phosphate-buffered saline (PBS), serially diluted, and plated for CFU numbers on LB plates containing antibiotics as indicated in figure axes and legends. Data are reported as either log CFU numbers recovered on particular antibiotic plates or as recombination efficiency, calculated as Gent^r^ CFU/Kan^r^ CFU. If no colonies were observed across triplicate plating, samples were listed as the detection limit, represented as if 0.5 colonies were counted. For recombination efficiency, the detection limit line is approximate, since the denominator (total CFU numbers) varies between samples.
